# Decreased Lymphangiogenic Activities and Genes Expression of Cord Blood Lymphatic Endothelial Progenitor Cells (VEGFR3^+^/Pod^+^/CD11b^+^ Cells) in Patient with Preeclampsia

**DOI:** 10.3390/ijms22084237

**Published:** 2021-04-19

**Authors:** Hayan Kwon, Ja-Young Kwon, Jeeun Song, Yong-Sun Maeng

**Affiliations:** 1 Department of Obstetrics and Gynecology, Institute of Women’s Life Medical Science, Yonsei University Health System, Seoul 03722, Korea; whitekwon@yuhs.ac (H.K.); jaykwon@yuhs.ac (J.-Y.K.); 2 Department of Obstetrics and Gynecology, Yonsei University College of Medicine, Seoul 03722, Korea; jsong82@jhmi.edu; 3 Program in Biochemistry, Cellular & Molecular Biology, Johns Hopkins University School of Medicine, Baltimore, MD 21205, USA

**Keywords:** lymphatic endothelial progenitor cells, lymphangiogenesis, differentiation, preeclampsia

## Abstract

The abnormal development or disruption of the lymphatic vasculature has been implicated in metabolic and hypertensive diseases. Recent evidence suggests that the offspring exposed to preeclampsia (PE) in utero are at higher risk of long-term health problems, such as cardiovascular and metabolic diseases in adulthood, owing to in utero fetal programming. We aimed to investigate lymphangiogenic activities in the lymphatic endothelial progenitor cells (LEPCs) of the offspring of PE. Human umbilical cord blood LEPCs from pregnant women with severe PE (*n* = 10) and gestationally matched normal pregnancies (*n* = 10) were purified with anti-vascular endothelial growth factor receptor 3 (VEGFR3)/podoplanin/CD11b microbeads using a magnetic cell sorter device. LEPCs from PE displayed significantly delayed differentiation and reduced formation of lymphatic endothelial cell (LEC) colonies compared with the LEPCs from normal pregnancies. LECs differentiated from PE-derived LEPCs exhibited decreased tube formation, migration, proliferation, adhesion, wound healing, and 3D-sprouting activities as well as increased lymphatic permeability through the disorganization of VE-cadherin junctions, compared with the normal pregnancy-derived LECs. In vivo, LEPCs from PE showed significantly reduced lymphatic vessel formation compared to the LEPCs of the normal pregnancy. Gene expression analysis revealed that compared to the normal pregnancy-derived LECs, the PE-derived LECs showed a significant decrease in the expression of pro-lymphangiogenic genes (*GREM1*, *EPHB3*, *VEGFA*, *AMOT*, *THSD7A*, *ANGPTL4*, *SEMA5A*, *FGF2*, and *GBX2*). Collectively, our findings demonstrate, for the first time, that LEPCs from PE have reduced lymphangiogenic activities in vitro and in vivo and show the decreased expression of pro-lymphangiogenic genes. This study opens a new avenue for investigation of the molecular mechanism of LEPC differentiation and lymphangiogenesis in the offspring of PE and subsequently may impact the treatment of long-term health problems such as cardiovascular and metabolic disorders of offspring with abnormal development of lymphatic vasculature.

## 1. Introduction

Preeclampsia (PE), a complication associated with 5–8% of pregnancies worldwide, is a hypertensive disease induced during pregnancy that is associated with systemic vasculopathy and multi-organ failure. It is known that long-term abnormalities in the fetus in utero may contribute to the subsequent development of adulthood disease in the offspring [1,2], and recent epidemiologic studies have indicated that the offspring of preeclamptic mothers display altered disease susceptibility [3]. Previous studies have demonstrated that the offspring of preeclamptic mothers have a higher risk of cardiovascular disease [4,5], hypertension [6,7], stroke [8], metabolic disturbances such as diabetes [9,10] and obesity [11,12,13], as well as increased susceptibility to inflammatory conditions [14] such as asthma [15,16].

The mechanism by which in utero fetal exposure to PE results in the reprograming of fetal physiology and contributes to disease susceptibility is largely unknown. However, several studies have suggested that in utero fetal programming that compromises the intrauterine environment may permanently alter the structure and function of the biological system of the fetus, ultimately increasing the individual’s susceptibility to disease later in life. Previous studies have reported epigenetic modifications and/or altered gene expression in the fetal endothelial progenitor cells (EPCs) exposed to PE in utero. These changes were associated with diminished proliferation, increased senescence, or abnormal endothelial cell function [17,18,19,20,21]. Therefore, changes in cellular behavior that culminate in dysfunctional angiogenesis may serve as one of the possible mechanisms underlying compromised in utero programming in the case of severe PE [22,23,24,25].

The lymphatic vasculature is a network of vessels formed by the lymphatic endothelial cells (LECs), and is traditionally known to maintain fluid homeostasis, absorb lipids from the intestine, and transport immune and antigen-presenting cells from inflammatory tissues to lymph nodes [26,27]. Growing evidence suggests the involvement of the lymphatic system in the modulation of the inflammatory response [28]. The lymphatic channel regulates peripheral immune tolerance by facilitating the entry of antigens and antigen-presenting cells into the immune system, controlling immune cell trafficking, and inducing T cell tolerance [29,30,31,32]. In particular, abnormal or dysfunctional lymphangiogenesis has been implicated in chronic inflammatory disease [33,34,35], autoimmune diseases [34,36], and metabolic disorders such as diabetes and obesity [37,38].

Angiogenesis and lymphangiogenesis share many common angiogenic signaling pathways. Therefore, abnormal lymphangiogenesis is often reported in pathophysiologies with dysfunctional angiogenesis [39,40,41]. The pathophysiology of PE is associated with dysfunctional angiogenesis, including the upregulated expression of anti-angiogenic factors that induce systemic endothelial dysfunction, thereby result in hypertension, proteinuria, and other systemic manifestations. We have previously reported significant abnormal lymphatic development in the decidua of preeclamptic woman [20]. However, less is known about the adverse effect of PE on lymphangiogenesis in leading to an unhealthy lymphatic system.

Here, we hypothesized that exposure to PE may exert significant adverse effects on the lymphatic endothelial progenitor cells (LEPCs) of the offspring, leading to abnormal lymphangiogensis. The functional impairment in the LEPCs of the offspring of preeclamptic mothers may form part of the mechanism underlying in utero programming, potentially producing health effects later in adult life. We show that the potential of LEPCs to differentiate into LECs is reduced, and the lymphangiogenic function of LECs is significantly diminished, in PE both in vitro and in vivo. We discuss the potential role of differentially expressed lymphangiogenesis-associated genes in offspring’s health.

## 2. Results

### 2.1. Isolation and Characterization of Human Cord Blood-Derived VEGFR3^+^/Pod^+^/CD11b^+^ Cells as LEPCs and the Induction of Their Differentiation into LECs

**Figure 1:**
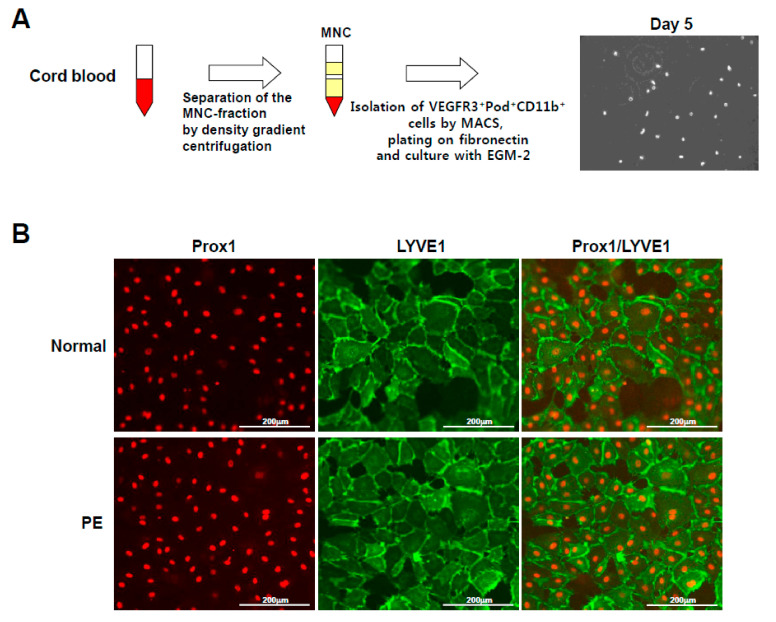
Isolation and characterization of the VEGFR3^+^/Pod^+^/CD11b^+^ cells. (**A**) The VEGFR3^+^/Pod^+^/CD11b^+^ cells were isolated by density-gradient centrifugation using Biocoll for 30 min at 400× *g*, washed thrice with phosphate-buffered saline, and purified by positive selection using VEGFR3^+^/Pod^+^/CD11b^+^ microbeads and a magnetic cell sorter device. (**B**) The VEGFR3^+^/Pod^+^/CD11b^+^ cells from preeclamptic (*n* = 10) and normal (*n* = 10) pregnancies were differentiated into ten LECs, respectively, and the expression of LEC-specific markers, Prox1 and LYVE1, was confirmed by immunofluorescence staining. MNC, mononuclear cell; MACS, magnetic-activated cell sorting; PE, preeclampsia; LEC, lymphatic endothelial cell.

To compare the lymphangiogenic functional capacity of LEPCs derived from PE and normal pregnancies, we isolated the vascular endothelial growth factor receptor 3 (VEGFR3)^+^/podoplanin (Pod)^+^/CD11b^+^ cells from the mononuclear cells of human cord blood via magnetic-activated cell sorting (Figure 1A). Upon culturing in EGM-2 in the presence of fibronectin, the VEGFR3^+^/Pod^+^/CD11b^+^ cells differentiated into LECs, which expressed the LEC-specific markers LYVE-1 and Prox1 (Figure 1B). These results demonstrate that VEGFR3^+^/Pod^+^/CD11b^+^ cells from cord blood can serve as LEPCs that can differentiate into LECs.

### 2.2. VEGFR3^+^/Pod^+^/CD11b^+^ LEPCs from Women with PE Show Diminished Differentiation into LECs

**Figure 2:**
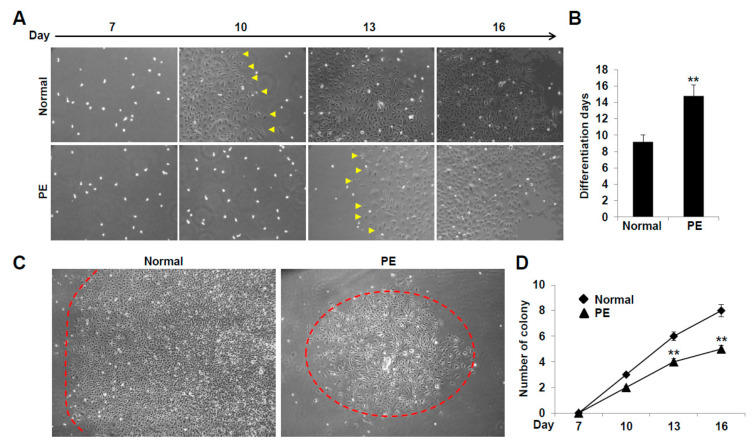
VEGFR3^+^/Pod^+^/CD11b^+^ lymphatic endothelial progenitor cells (LEPCs) derived from preeclamptic women show decreased differentiation potency. (**A**) Morphological assessment of (VEGFR3^+^/Pod^+^/CD11b^+^) LEPC differentiation into LECs upon culture with fibronectin (10 µg/mL). The arrowheads indicate the differentiated LEC colonies. Images were viewed at 100× magnification. (**B**) Quantitative analysis of the differentiation days. The data are presented as the mean ± standard error (SE). **, *p* < 0.05 vs. normal. (**C**) Morphological assessment of LEPC differentiation into LECs upon culture with fibronectin (10 µg/mL). The dashed-line circles indicate the differentiated LEC colonies. Images were viewed at 40× magnification. (**D**) Quantitative analysis of differentiated LEC colonies. All experiments were performed in ten different cell lines. The data are presented as the mean ± SE. **, *p* < 0.05 vs. normal. LEC, lymphatic endothelial cell; PE, preeclampsia.

We compared the differentiation ability of the LEPCs derived from woman with PE and those with normal pregnancy by investigating the time required for their differentiation into LECs and evaluating the number and size of differentiated LEC colonies. The VEGFR3^+^/Pod^+^/CD11b^+^ LEPCs from normal pregnancies differentiated into LECs after 9 days of culture (Figure 2A,B). However, it took 14 days for the VEGFR3^+^/Pod^+^/CD11b^+^ LEPCs from PE-complicated pregnancies to differentiate into LECs (Figure 2A,B). Moreover, both the number and size of the LEC colonies were decreased in the PE group compared with those in the normal pregnancy group on the same differentiation days (Figure 2C,D). These results suggest that the VEGFR3^+^/Pod^+^/CD11b^+^ LEPCs from woman with PE have a diminished differentiation capability compared to those from normal pregnancies.

### 2.3. LECs Derived from VEGFR3^+^/Pod^+^/CD11b^+^ LEPCs of PE Show Decreased Lymphangiogenic Activities In Vitro

**Figure 3:**
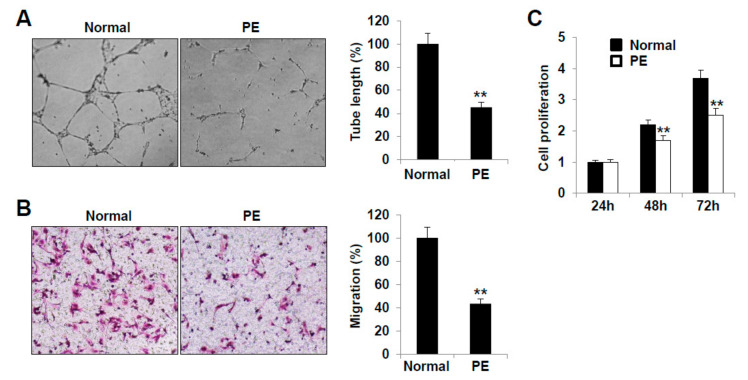
The lymphangiogenic functions of LECs derived from preeclamptic and normal pregnancies. (**A**) Tubular network formation of the LECs derived from normal and PE pregnancies on Matrigel was photographed and quantified at 24 h. (**B**) Migration was quantified by counting the cells that migrated to the lower surface of the filters via optical microscopy at 200× magnification. (**C**) LECs (1 × 10^3^ cells) were seeded into a 96-well plate in triplicate for each condition. At the end of the incubation, the supernatants were removed, and each well was treated with 150 µL DMSO. The absorbance value (OD) of each well was measured at 490 nm. All experiments were performed in triplicate with three different cell lines. LEC, lymphatic endothelial cell; DMSO, dimethyl sulfoxide; PE, preeclampsia. **, *p* < 0.01 vs. normal.

We next analyzed the in vitro lymphangiogenic activities of LECs differentiated from the VEGFR3^+^/Pod^+^/CD11b^+^ LEPCs from women with PE and those with normal pregnancies. The LECs were able to form capillary-like tubes on the Matrigel. The tube connection and network formation were decreased in the PE group compared to those of the normal pregnancy group. Furthermore, the length and area of the tubes formed by the PE group LECs were diminished compared to those of the normal pregnancy control LECs (Figure 3A).

In the migration assay, the number of transmigrated cells was significantly reduced in the PE group (Figure 3B). Furthermore, the proliferation activity of the LECs from the PE group was decreased compared to that of the LECs from the normal pregnancy group at 48 and 72 h (Figure 3C).

**Figure 4:**
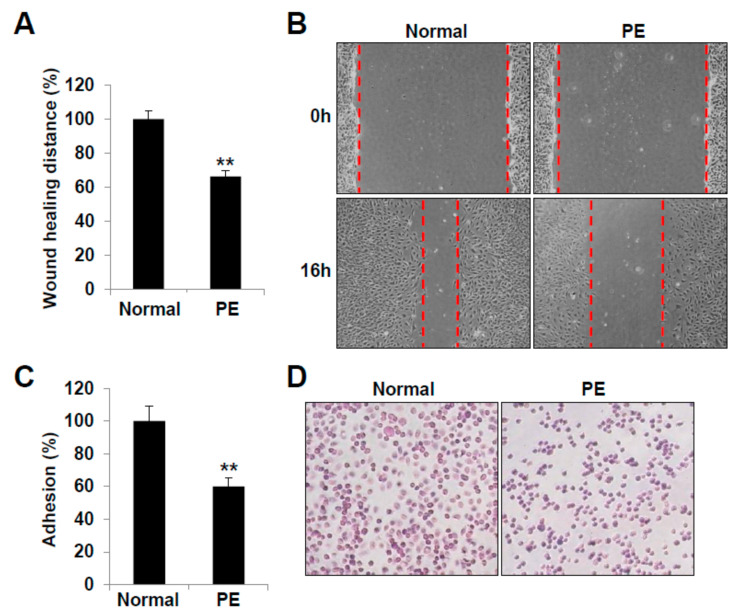
LECs derived from preeclamptic pregnancies show reduced wound healing and adhesion activity. (**A**,**B**) LECs were seeded on 35-mm dishes. The confluent cell layer was scratched using a micropipette tip and washed to remove any debris. Images were captured at 0 and 16 h after wounding. For quantitative analysis, five fields per plate were photographed, and the distances between the front lines were measured using ImageJ. (**C**,**D**) A 96-well plate was coated overnight (4 °C) with 0.1 mg/mL human fibronectin. LECs in an adhesion buffer were seeded at 10^5^ cells/well in 100-µL volume and incubated for 30 min at 37 °C. Cell adhesion was quantified by counting the cells that attached to the fibronectin-coated matrix using optical microscopy at 200× magnification. All data are presented as the mean ± standard error (SE) from three different cell lines performed in triplicate. **, *p* < 0.01 vs. normal. LEC, lymphatic endothelial cell; PE, preeclampsia.

After wounding, the maximal migration of the cells into the wounded area was reduced in the PE group LECs (Figure 4A). The remnant wound area after 16 h was larger for the PE group than for the normal pregnancy group (Figure 4B). Moreover, the LECs from the PE group showed a reduced adhesion activity (Figure 4C,D). These results indicate that LECs differentiated from VEGFR3^+^/Pod^+^/CD11b^+^ LEPCs of PE have decreased lymphangiogenic activities in vitro and may be associated with the abnormal development of lymphatic vessels in the offspring of PE.

### 2.4. LECs Differentiated from VEGFR3^+^/Pod^+^/CD11b^+^ LEPCs of PE Present Reduced 3D Lymphatic Sprouting

**Figure 5:**
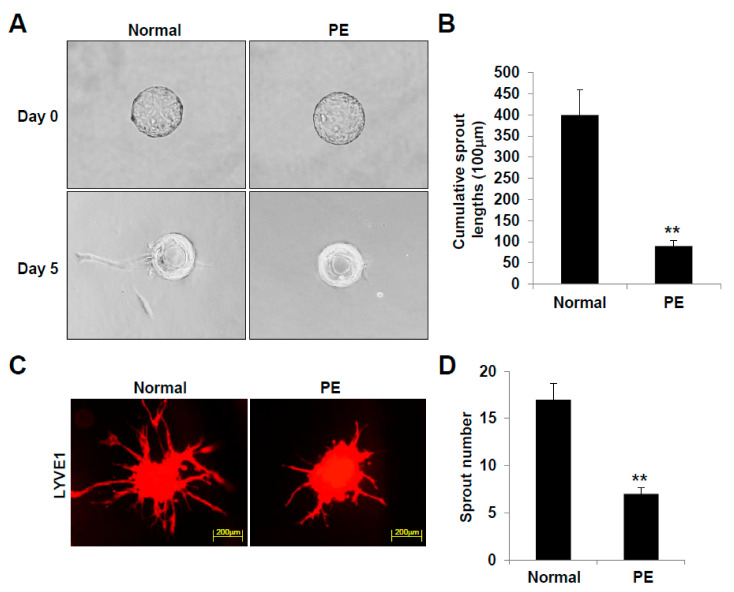
LECs derived from preeclamptic pregnancies show suppressed lymphatic vessel sprouting and lymphangiogenic function in vitro. (**A**) In vitro 3D lymphangiogenesis with fibrin gel-embedded microbeads of LECs. (**B**) The cumulative length of all sprouts originating from an individual spheroid was quantified after 5 days. **, *p* < 0.01 vs. normal. (**C**) LEC spheroids were embedded in 20% Matrigel-containing fibrin gels and cultured for 48 h. Spheroids were stained for PECAM-1. (**D**) Quantification of sprout number. All data are presented as the mean ± standard error (SE) from three different cell lines with *n* = 7 per group per experiment. **, *p* < 0.01 vs. normal. LEC, lymphatic endothelial cell; PE, preeclampsia.

We next compared the sprouting ability of the LECs differentiated from LEPCs from preeclamptic and normal pregnancies. After 5 days of culture, the cumulative sprout length of the 3D-bead LECs differentiated from the preeclamptic pregnancy-derived VEGFR3^+^/Pod^+^/CD11b^+^ LEPCs was significantly reduced compared to that of the LECs obtained from the normal pregnancy-derived LEPCs (Figure 5A,B). In addition, the number of sprouts emerging from the LECs of the PE group was also decreased in the spheroid sprouting assay (Figure 5C,D). These observations provide evidence that LECs differentiated from VEGFR3^+^/Pod^+^/CD11b^+^ LEPCs of PE exhibit disrupted lymphangiogenic activity.

### 2.5. LECs Differentiated from VEGFR3^+^/Pod^+^/CD11b^+^ LEPCs of PE Show Increased Lymphatic Vessel Permeability

**Figure 6:**
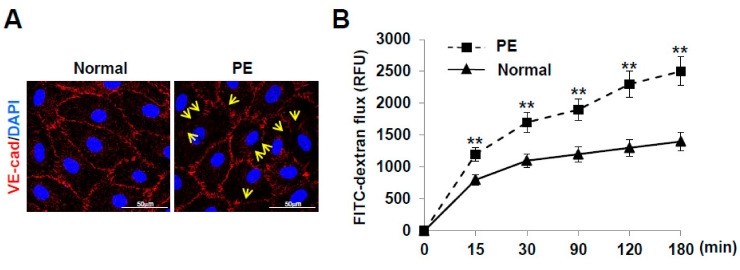
LECs derived from preeclamptic pregnancies show increased permeability through the disorganization of lymphatic VE-cadherin junctions. (**A**) Confluent LECs were fixed and stained with anti-VE-cadherin antibody. The arrows indicate the disruption of the VE-cadherin junction at the plasma membrane. (**B**) FITC-dextran permeability was evaluated. The experiments were performed in triplicate with three different cell lines. The data are presented as the mean ± standard error (SE). **, *p* < 0.01 vs. normal. PE, preeclampsia.

VE-cadherin is known to mediate the endothelial adhesion required to form intercellular junctions and preserve lymphatic vessel structure and integrity. We investigated whether the localization of VE-cadherin in cell–cell junctions was altered in LECs of PE compared to normal. In the LECs derived from normal pregnancies, VE-cadherin was detected at the plasma membrane in a linear pattern at the cell border. In contrast, the LECs derived from preeclamptic pregnancies displayed decreased expression of VE-cadherin at the plasma membrane, and a disrupted linear pattern (Figure 6A). In addition, compared to the normal pregnancy-derived LECs, the preeclamptic pregnancy-derived LECs showed an increase in FITC-dextran permeability in a time-dependent manner (Figure 6B). These results indicate that LECs derived from VEGFR3^+^/Pod^+^/CD11b^+^ LEPCs of PE show increased lymphatic permeability by disorganizing lymphatic VE-cadherin junctions.

### 2.6. VEGFR3^+^/Pod^+^/CD11b^+^ LEPCs from Women with PE Show Decreased Lymphangiogenic Activity In Vivo

**Figure 7:**
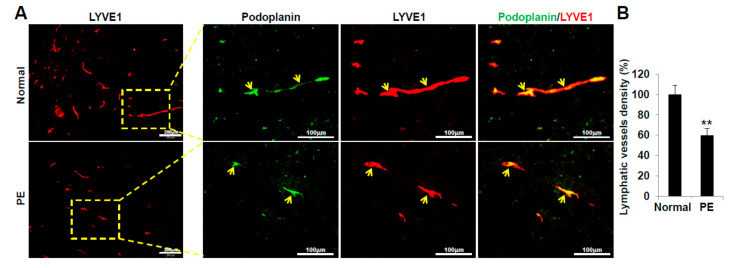
VEGFR3^+^/Pod^+^/CD11b^+^ lymphatic endothelial progenitor cells (LEPCs) derived from preeclamptic pregnancies show decreased LEC differentiation and lymphatic vessel formation in vivo. (**A**) Seven-week-old male C57/BL6 mice were subcutaneously injected with 0.6 mL Matrigel containing 5 × 10^4^ human LEPCs. After 14 days, the plugs were removed and washed with PBS. Plug sections were used to identify the differentiated LEPCs using anti-LYVE1 (green) and anti-podoplanin (red) antibodies. (**B**) Quantitative assessment of the LYVE1- and CD31-positive lymphatic vessels per field for each section. The experiments were performed in triplicate with three different cell lines. The data are presented as the mean ± standard error (SE). **, *p* < 0.01 vs. normal. PE, preeclampsia. Scale bar, 200 μm, 100 μm.

Next, we assessed the differentiation activity and lymphatic vessel formation of VEGFR3^+^/Pod^+^/CD11b^+^ LEPCs from PE and normal pregnancy in vivo. Matrigel containing human VEGFR3^+^/Pod^+^/CD11b^+^ LEPCs was subcutaneously injected into C57/BL6 mice. After 14 days, the plugs were sectioned and stained for human LEC- and lymphatic vessel-specific markers, podoplanin and LYVE-1, respectively. The PE-derived LEPCs showed reduced differentiation activity into LECs and a significantly lower density of podoplanin/LYVE1-positive lymphatic vessels compared to the normal pregnancy-derived LEPCs (Figure 7A). Quantification of podoplanin/LYVE-1-positive lymphatic structures confirmed that vessel density was significantly reduced in PE-derived LEPCs as compared with normal (Figure 7B). Overall, our results indicate that PE-derived LEPCs exert diminished lymphangiogenic activity in vivo.

### 2.7. Gene Expression Analysis of LECs Derived from LEPCs of Preeclamptic Women

**Figure 8:**
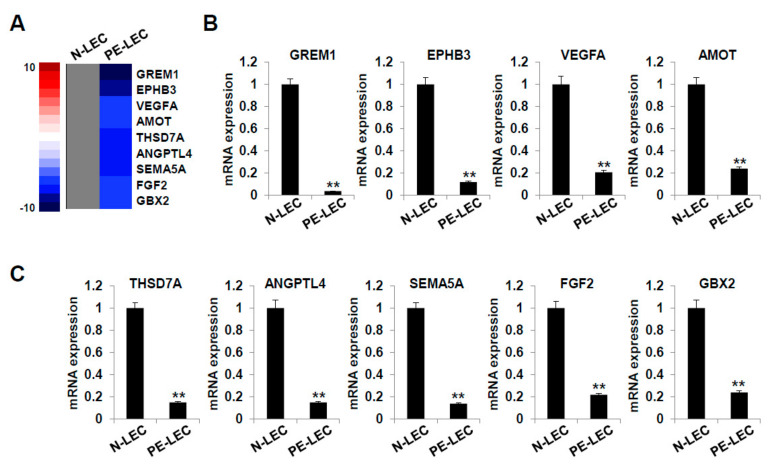
RNA-sequencing for gene expression analysis using LECs derived from normal and preeclamptic pregnancies. (**A**) Cluster of downregulated (four-fold) lymphangiogenesis-related genes in preeclamptic pregnancy-derived LECs (*n* = 3) versus normal LECs (*n* = 3). (**B**,**C**) RT-qPCR analysis of the lymphangiogenesis-related genes. Gene expression was normalized to *GAPDH*. N-LEC, normal pregnancy-derived lymphatic endothelial cell; PE-LEC, the preeclamptic pregnancy-derived lymphatic endothelial cell. **, *p* < 0.01 vs. N-LEC.

We next used RNA-sequencing to investigate the changes in gene expression related to the dysfunctional lymphangiogenesis observed in PE. As shown in Figure 8A, the expression of *GREM1*, *EPHB3*, *VEGFA*, *AMOT*, *THSD7A*, *ANGPTL4*, *SEMA5A*, *FGF2*, and *GBX2* was significantly down-regulated in the LECs differentiated from the preeclamptic pregnancy-derived LEPCs. The levels of these genes were further confirmed by RT-qPCR and found to be significantly down-regulated in the LECs derived from preeclamptic pregnancies (Figure 8B,C). These results suggest that the down-regulated expression of various lymphangiogenesis-related genes may be related to the decreased lymphangiogenic function of LECs from preeclamptic pregnancy.

## 3. Discussion

PE is known to exert adverse long-lasting effects on the offspring and contribute to cardiovascular, metabolic, and neurological diseases. However, the underlying mechanism is poorly understood. The most promising hypothesis is that suboptimal intrauterine environments during pregnancy with PE may permanently alter the organ structure and biological feedback mechanism of the fetus, increasing the individual’s susceptibility to disease [42,43], and that stem cells may be involved in the influence of the in utero environment on the subsequent risk of disease onset in adult life [44,45].

**Figure 9:**
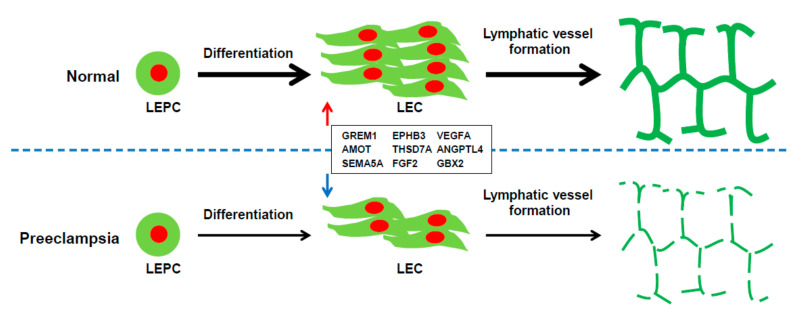
Proposed model describing the differentiation of LEPCs to LECs under preeclamptic and normal pregnancy conditions. The differentiation of the cord blood-derived LEPCs to LECs was significantly reduced under preeclamptic pregnancy conditions compared to normal pregnancy conditions. Moreover, the LECs differentiated from the preeclamptic pregnancy-derived LEPCs displayed decreased lymphatic vessel formation activity. Dysfunctional lymphangiogenesis in preeclamptic pregnancy-derived cells was associated with the downregulated expression of pro-lymphangiogenic genes.

The present study demonstrates that in utero exposure to PE is associated with the decreased differentiation of cord blood-derived LEPCs into LECs, as well as the impaired lymphangiogenic functions of LECs in vitro and in vivo. In addition, dysfunctional lymphangiogenesis in PE was associated with the downregulated expression of pro-lymphangiogenic genes (Figure 9). These findings indicate the formation of an abnormal lymphatic system in the offspring exposed to PE, which may then be associated with the adverse health conditions of the offspring later in life.

Lymphangiogenesis, that is the formation of new lymphatic vessels from preexisting ones, involves the proliferation, migration, and tube formation of LECs. Growing evidence has highlighted the contribution of abnormal lymphangiogenesis to the pathogenesis of cardiovascular, metabolic, and neurodevelopmental diseases that are common in offspring exposed to PE. For example, Viven et al. reported severely impaired cardiac regeneration in the absence of a functional lymphatic network in adult zebrafish hearts. These authors revealed an association between the absence of a functional lymphatic vascular system and cardiac hypertrophy, metabolic disruption, and inflammation [46]. Moreover, Yuuki et al. demonstrated that lymphangiogenesis is related to ischemia-induced heart failure and reperfusion. They found that inhibition of the endogenous lymphangiogenesis response could exacerbate ischemia-induced heart failure [47]. Lymphatic malfunction also contributes to adult-onset obesity [48,49]. Harvey et al. revealed that compromised lymphatic vascular integrity was the cause of the excessive accumulation of fat in Prox1^+/−^ mice, which became progressively obese with age [49]. In relation to neurodevelopmental diseases, malfunction of the lymphatic vessel has been associated with autism spectrum disorder, which is more common in offspring exposed to PE [50,51]. In light of these previous reports, the present study provides important evidence that PE is a critical factor related to dysfunctional lymphangiogenesis in offspring, mediated via a reduction in the lymphangiogenic capacity and function of LEPCs and LECs, which may substantially affect the long-term health of offspring.

We demonstrated that the dysfunctional lymphangiogenesis in PE is associated with the downregulated expression of lymphangiogenesis-related genes, including *GREM1*, *EPHB3*, *VEGFA*, *AMOT*, *THSD7A*, *ANGPTL4*, *SEMA5A*, *FGF2*, and *GBX2*. Previous studies have highlighted the important functions of these genes. These genes are closely associated with angiogenesis and lymphangiogenesis, and differential expression may alter the function and subsequently affect disease susceptibility such as cardiovascular and metabolic disease.

Gremlin 1 (GREM1) binds to VEGFR2 and promotes the proliferation, migration, and angiogenic sprout formation of endothelial cells by tyrosine phosphorylation and subsequent activation of downstream molecules [52]. The gremlin-VEGFR2 axis was reported to be closely associated with angiogenesis in both physiological and pathological processes [53]. In addition, knockdown of GREM1 suppresses cell growth and angiogenesis [54]. 

Ephrin type-B receptor 3 (EPHB3) is critically important for the remodeling of the embryonic vascular system; it controls the migration, adhesion, and sprouting behavior of endothelial cells in vitro in a similar way as angiopoietin 1 (Ang1) and VEGF [55]. EPHB3 is also involved in immune cell activation and trafficking and is related to the pathogenesis of various diseases, including immune-mediated conditions, cancer, atherosclerosis, and central nervous system diseases [56]. Moreover, EPHB3 expressed in vascular endothelial cells regulates angiogenesis in peripheral nerve injury [57].

Down regulation of VEGFA (vascular endothelial growth factor A) inhibits angiogenesis and lymphangiogenesis [58]. VEGFA promotes lymphangiogenesis and high endothelial venule expansion in lymph nodes [59]. K14 Vegf-A Tg Vegfr1 tk(−/−) mice exhibit a remarkable decrease in lymphangiogensis as well as angiogenesis in subcutaneous tissues [60].

AMOT (angiomotin) has been shown to regulate endothelial cell migration during embryonic angiogenesis [61,62]. Anti-AMOT antibodies significantly decreased the number of endothelial filopodia and inhibited vessel migration during retinal angiogenesis in vivo [63]. DNA vaccine targeting AMOT also inhibits angiogenesis and tumor growth [64].

Thrombospondin type I domain-containing 7A (THSD7A) promotes endothelial cell migration and tube formation in angiogenesis [65,66]. Knockdown of Thsd7a disrupted angiogenic sprouting via Notch-dll4 signaling during zebrafish development [67,68].

Angiopoietin like 4 (ANGPTL4) is known as the fasting-induced adipose factor (FIAF) and is related to lymphatic endothelial function. LEC functions were severely reduced in mice without ANGPTL4 expression, and ANGPTL4 mutants showed dilated and blood-filled lymphatics, which were aberrantly connected to blood vessels [69]. ANGPTL4 also promotes angiogenesis in a mouse model of acute ischemic stroke [70].

SEMA5A, a member of the semaphoring V subfamily, increases endothelial cell migration, proliferation and promotes angiogenesis [71]. Null mutation of SEMA5A results in defective remodeling of the cranial vascular system in mice [72].

FGF2, also known as basic fibroblast growth factor (bFGF), regulates lymphangiogenesis and vascular permeability [73,74], and induces corneal blood and lymphatic vessel growth [75]. Reciprocal interplay between FGF-2 and VEGF-C collaboratively stimulated angiogenesis, intratumoral lymphangiogenesis, tumor growth, and metastasis [76]. In addition, FGF2 promotes vegf-c dependent lymphangiogeneis and knockdown of FGF2 inhibited lymphangiogenesis in vitro and in vivo [77].

GBX2 (gastrulation brain homeobox 2) gene silencing has an inhibition effect on invasion, proliferation and angiogenesis by inhibiting the activation of the Wnt/β-catenin signaling pathway in breast cancer cells [78].

These results suggest that the downregulated expression of various lymphangiogenesis-related genes may be related to the decreased function of LECs derived from preeclamptic pregnancies. Changes in the expression of these genes in the umbilical cord blood from PE may be the basis for explaining why offspring of women with PE during pregnancy are more likely to develop various diseases. However, the detailed mechanism of how the downregulation of these lymphangiogenesis-related genes affects the function of preeclamptic pregnancy-derived LECs remains to be resolved.

To our knowledge, this is the first study to demonstrate the impaired functions and dysregulated lymphangiogenesis of LEPCs derived from cord blood of preeclamptic pregnancies in vitro and in vivo. Our study also improves the current understanding of the fetal origin of disease in response to PE and in utero programming. While the majority of studies on dysfunctional lymphangiogenesis focus on cancer metastasis, the current study suggests the plausible involvement of lymphangiogenesis in the pathogenesis of chronic diseases such as cardiovascular and metabolic disorders. We also provide information about potential therapeutic targets for long-term PE-related complications in mothers and offspring.

## 4. Materials and Methods

### 4.1. Study Population and Sample Collection

We enrolled subjects that delivered either vaginally or by cesarean section at 36–41 weeks of gestation at Severance Hospital between September 2016 and July 2017. The umbilical cord blood was obtained for VEGFR3^+^/Pod^+^/CD11b^+^ LEPC isolation at the time of delivery after fetal expulsion from women with PE (*n* = 10) and those with normal pregnancies (*n* = 10) (Table 1). The women assigned to the “PE” group exhibited severe PE comprising hypertension (defined as a blood pressure level >160/110 mmHg on two occasions at least 4 h apart during bed rest) in association with thrombocytopenia (indicated by a platelet count <100,000/µL), impaired liver function (indicated by a 2× elevation from normal levels of liver enzyme blood concentrations), severe persistent right-upper quadrant or epigastric pain that was unresponsive to medication and/or not accounted for by an alternative diagnosis, progressive renal insufficiency (indicated by a serum creatinine concentration >1.1 mg/dL or a 2× elevation in the serum creatinine concentration in the absence of any other renal disease), pulmonary edema, and/or new-onset cerebral and/or visual disturbances, as per the American College of Obstetricians and Gynecologists guidelines [79,80]. Pregnancies associated with premature membrane rupture, fetal malformation, chromosome anomalies, multiple pregnancies, and/or renal or endocrine diseases other than diabetes mellitus were excluded from the study. All patients provided informed consent before their participation in the study, which was approved by the Institute Review Board of Severance Hospital (4-2016-1022) on 12 January 2017.

### 4.2. Isolation and Cultivation of LEPCs (VEGFR3^+^/Pod^+^/CD11b^+^ Cells)

Mononuclear cells were isolated from human umbilical cord blood samples (approximately 50 mL each) by density-gradient centrifugation using Biocoll (Biochrom, Berlin, Germany) for 30 min at 400× *g*. The samples were washed thrice in phosphate-buffered saline (PBS; Biochrom), and the VEGFR3^+^/Pod^+^/CD11b^+^ cells were purified by positive selection using anti-VEGFR3/Pod/CD11b microbeads (Miltenyi Biotec, Bergisch-Gladbach, Germany) and a magnetic cell sorter device (Miltenyi Biotec). The purity was >98%, as assessed by fluorescence-activated cell sorting (FACS). The VEGFR3^+^/Pod^+^/CD11b^+^ cells were seeded into six-well plates coated with human fibronectin (Sigma, St. Louis, MO, USA) in endothelial basal medium-2 (EBM-2; Clonetics, Cell Systems, St. Katharinen, Germany). The medium was supplemented with endothelial growth medium-2 (EGM-2; Clonetics, Cell Systems) containing fetal bovine serum (FBS), human VEGF-A, human fibroblast growth factor-B, human epidermal growth factor, insulin-like growth factor 1 (IGF1), and ascorbic acid at the recommended concentrations. Cultures were maintained for 7 days, and phenotypic analyses of the cells were performed on day 3, 5, and 7.

### 4.3. LEPC Differentiation Assay

LEPCs (5 × 10^5^ cells/well, *n* = 10) were seeded in six-well plates coated with fibronectin (10 µg/mL; Sigma) and cultured in EGM-2. The medium was replaced every 2 days, and the morphological assessment of LECs was performed using light microscopy. The differentiation day was calculated as the first day on which a differentiated LEC colony was observed from the time of LEPC seeding. The differentiation day and number of colonies formed in each set were determined using light microscopy. Ten cell lines were used for the experiment.

### 4.4. In Vitro Tube Formation Assay

Tube formation was assayed as previously described [81]. Matrigel solution (250 µL) (BD Biosciences, Bedford, MA, USA) was added to a 16 mm-diameter tissue culture well and allowed to polymerize (30 min, 37 °C). LECs (1.2 × 10^5^ cells/well) were resuspended in EBM and plated onto Matrigel. Matrigel cultures were incubated at 37 °C and cells were photographed at 16 and 24 h of incubation (200× magnification). The mature tube network was determined by scanning photographs of the tubes into Adobe Photoshop. ImageJ software (National Institute of Health) was used to quantify the identified areas.

### 4.5. Cell Proliferation Assay

LECs (1 × 10^3^ cells) were seeded into a 96-well plate. The cells were cultured for 72 h and incubated with 20 µL of the 3-(4,5-dimethylthiazol-2-yl)-2,5-diphenyl tetrazolium bromide (MTT; 5 mg/mL) reagent. After 4 h, the supernatants were removed, and the cells were treated with 150 µL dimethyl sulfoxide (DMSO). The absorbance value (OD) of each well was measure at a wavelength of 490 nm. The experiments were performed in triplicate with three different cell lines.

### 4.6. Cell Migration Assay

Cell migration was assayed using the Transwell system (Corning Costar, Acton, MA, USA) using 6.5 mm-diameter polycarbonate filters (8-µm pores). The lower surface of the filter was coated with 10 µg/mL fibronectin (Sigma-Aldrich, St. Louis, MO, USA), and LECs (10^5^ cells) from passage 3 were seeded onto chemotaxis filters in EBM supplemented with 0.5% FBS. After 5 h incubation, non-migrating cells were removed and migrating cells were analyzed by hematoxylin and eosin (H&E) staining. Quantification was performed using Kodak 1D software (Eastman Kodak, Rochester, NY, USA). The results were performed in triplicate with three different cell lines.

### 4.7. Wound Healing Assay

The wound healing was performed by scratching a confluent layer of LECs on 35-mm dishes using a micropipette tip. The cells were then washed and captured at 0 and 16 h. For quantitative analysis, five fields per plate were photographed, and the distances between the front lines were measured using ImageJ software (National Institutes of Health, Bethesda, MD, USA). Each assay was performed in triplicate with three different cell lines.

### 4.8. Cell Matrix Adhesion

The cell matrix adhesion was performed as previously described [82]. 96-well plates were coated with 10 µg/mL human fibronectin overnight at 4 °C (Sigma-Aldrich, St. Louis, MO, USA). Then, 10^5^ LECs were seeded and incubated for 30 min at 37 °C (100 µL adhesion buffer comprising serum-free medium and EBM). After the nonadherent cells were discarded, the adherent cells were stained with H&E. Quantification was performed in triplicate by counting the adherent cells in five randomly selected fields per well (Axiovert 100; Carl Zeiss Micro-Imaging, Thornwood, NY, USA). The results were performed in triplicate with three different cell lines.

### 4.9. Three-Dimensional (3D) Bead Sprouting Assay

LECs were coated on Cytodex 3 microcarrier beads (ratio of 400 cells/bead: GE Healthcare Bio-Sciences Corp, Piscataway, NJ, USA) in Microvascular Endothelial Cell Growth Medium-2 MV (Lonza) and embedded in 2 mg/mL fibrin gels in 48-well plates by mixing 2 mg/mL fibrinogen (Chemicon Inc., Rolling Meadow, IL, USA) in HBSS, 1 U/mL thrombin (Sigma-Aldrich, St. Louis, MO, USA), and 150 µg/mL aprotinin (Sigma-Aldrich, St. Louis, MO, USA). WI-38 cells (11,000 cells/well) were cultured for 5 days by changing the medium every other day. Sprouted LECs were fixed with 4% paraformaldehyde for 1 h at room temperature (20 to 25 °C). Bright-field images were captured using Axiovert 200 (Zeiss) at 5× magnification, and sprout lengths were measured using ImageJ software.

### 4.10. Three-Dimensional (3D) Spheroid Sprouting Assay

LECs (3000 cells) were cultured in round-bottom 96-well plates (Nunc, Rochester, NY, USA) precoated with 0.8% agarose for 1 day and the spheroids were collected and embedded in 20% Matrigel-containing (BD Biosciences) fibrin gels (3 mg/mL fibrinogen, 2 U/mL thrombin, and 200 µg/mL aprotinin). The spheroids were cultured for 48 h, fixed with 4% paraformaldehyde for 1 h at room temperature, and stained with rabbit anti-human lymphatic vessel endothelial hyaluronan receptor 1 (LYVE1) polyclonal antibody (Abcam, Inc., Cambridge, MA, USA).

### 4.11. Immunofluorescence Staining

Confluent LECs were fixed with 3.7% formaldehyde for 20 min and permeabilized with 0.1% Triton X-100 in PBS. The LECs were then preincubated with a blocking solution of PBS containing 5% normal donkey serum and 0.05% Tween-20. The cells were incubated with goat anti-VE-cadherin polyclonal antibody (1:100 dilution, Santa Cruz Biotechnology Inc., Santa Cruz, CA, USA) for 2 h at room temperature (20 to 25 °C) and labeled with a fluorescein-conjugated secondary antibody (Molecular Probes, Eugene, OR, USA). The samples were observed under a fluorescence microscope (Olympus, Tokyo, Japan).

### 4.12. Transwell Permeability Assay

LECs were plated onto a Transwell filter (Corning, pore size: 3.0 µm, membrane diameter: 6.5 mm) and cultured for two days. The lower chamber was filled with 0.6 mL EBM, and serum-free EBM supplemented with 1 mg/mL fluorescein isothiocyanate (FITC)-dextran (average MW ~40,000) was added to the top chamber. A 50-µL aliquot of EBM was taken from the lower chamber at various time points, and the amount of FITC-dextran diffused to the bottom chamber was measured using a fluorometer (FluorStar Optima, BMG Labtech, Mornington, VIC, Australia) excitation: 485 nm; emission: 520 nm). The experiments were performed in triplicate with three different cell lines.

### 4.13. LEC Differentiation Assay in Matrigel

Seven-week-old male C57/BL6 mice (Orient Company, Seoul, Korea) were subcutaneously injected with 0.6 mL Matrigel containing 1 × 10^5^ human LEPCs. After 14 days, the plugs were removed and washed with PBS. Then, 8 µm-thick frozen Matrigel sections were blocked with 5% normal donkey serum in PBS containing 0.1% Triton X-100 and incubated overnight with a primary antibody (anti-human LYVE1 (Abcam, Inc., Cambridge, MA, USA) or anti-human podoplanin (R&D Systems, Minneapolis, MN, USA)) at 4 °C and labeled with a fluorescein-conjugated secondary antibody (Molecular Probes). The samples were observed under a fluorescence microscope (Olympus). The numbers of LYVE1 and podoplanin double-positive differentiated LEPCs and lymphatic vessels within the Matrigel were counted in six fields per section.

### 4.14. RNA-Sequencing for Gene Expression Analysis

Total RNA was isolated using Trizol reagent (Invitrogen), and RNA quality was assessed on an Agilent 2100 Bioanalyzer using the RNA 6000 Nano Chip (Agilent Technologies, Amstelveen, The Netherlands). RNA quantification was performed using an ND-2000 Spectrophotometer (Thermo Fisher Scientific Inc., Wyman Street Waltham, MA, USA).

For normal (*n* = 3) and preeclamptic (*n* = 3) pregnancy-derived LEC RNAs, library construction was performed using QuantSeq 3′-mRNA-Seq Library Prep Kit (Lexogen, Inc., Wien, Austria) according to the manufacturer’s instructions. In brief, 500 ng total RNA was prepared, hybridized with an oligo-dT primer containing an Illumina-compatible sequence at its 5′-end, and subjected to reverse transcription. After degradation of the RNA template, second-strand synthesis was initiated using a random primer containing an Illumina-compatible linker sequence at its 5′-end. The double-stranded library was purified using magnetic beads to remove all reaction components. The library was amplified to add the complete adapter sequences required for cluster generation. The finished library was purified from PCR components, and high-throughput sequencing was performed as single-end 75 sequencing using NextSeq 500 (Illumina, Inc., San Diego, CA, USA).

QuantSeq 3′-mRNA-Seq reads were aligned using Bowtie2 [83]. The Bowtie2 indices were either generated from the genome assembly sequence or the representative transcript sequence to align the genome and transcriptome. The alignment file was used to assemble the transcripts, estimate their abundances, and detect differential gene expression. Differentially expressed genes were determined based on the counts from unique and multiple alignments using coverage in BEDtools [84]. The read count (RC) data were processed based on the quantile normalization method using EdgeR within R [85] in Bioconductor [86]. Gene classification was based on searches performed using DAVID (http://david.abcc.ncifcrf.gov/, accessed on 21 July 2018) and the Medline database (http://www.ncbi.nlm.nih.gov/, accessed on 21 July 2018).

### 4.15. Real-Time Reverse-Transcription Quantitative Polymerase Chain Reaction (RT-qPCR)

Total RNA was isolated from the LECs derived from normal and preeclamptic pregnancies using Trizol reagent (Invitrogen, Carlsbad, CA, USA). Power SYBR Green RNA-to-CT^TM^ 1-Step kit (Applied Biosystems, Foster City, CA, USA) and StepOnePlus^TM^ (Applied Biosystems) were used to measure the mRNA expression of human genes according to the manufacturer’s instructions. PCR conditions were as follows: 48 °C for 30 min and 95 °C for 10 min, followed by 40 cycles of 95 °C for 15 s and 55 °C for 1 min. The results were based on the cycle threshold (Ct) values. Differences between the Ct values for the experimental and reference (*GAPDH*) genes were calculated, and the results are expressed as the ratio of each RNA level to the calibrated sample. The primers used for gene amplification are shown in Table 2.

### 4.16. Statistical Analyses

All experiments were performed in triplicate with three different cell lines in each group, and the data are presented as the mean ± standard error (SE). The statistical comparison between groups was performed by one-way analysis of variance (ANOVA) followed by Tukey’s test.

## 5. Conclusions

In conclusion, the present study demonstrates that LEPCs from PE displayed significantly delayed differentiation and reduced formation of lymphatic endothelial cell (LEC) colonies compared with the LEPCs from normal pregnancies. LECs from PE-derived LEPCs exhibited decreased proliferation, adhesion, tube formation, migration, wound healing, and 3D-sprouting activities compared with the normal pregnancy-derived LECs. LECs derived from preeclamptic pregnancies also show increased permeability through the disorganization of lymphatic VE-cadherin junctions. In vivo, LEPCs from PE showed significantly reduced LEC differentiation and lymphatic vessel formation compared to the LEPCs of the normal pregnancy.

Gene expression analysis revealed that compared to the normal pregnancy-derived LECs, the PE-derived LECs showed a significant decrease in the expression of pro-lymphangiogenic genes (*GREM1*, *EPHB3*, *VEGFA*, *AMOT*, *THSD7A*, *ANGPTL4*, *SEMA5A*, *FGF2*, and *GBX2*). Overall, our findings demonstrate, for the first time, that LEPCs from PE have reduced differentiation and lymphangiogenic activities in vitro and in vivo and show decreased expression of pro-lymphangiogenic genes. This study opens a new avenue for investigation of the molecular mechanism of LEPC differentiation and lymphangiogenesis in the offspring of PE and subsequently may impact the treatment of long-term health problem such as cardiovascular and metabolic disorders of offspring with abnormal development of lymphatic vasculature.

## Figures and Tables

**Table 1:** Patient characteristics.

	Normal Pregnancy (*n* = 10)	Preeclampsia (*n* = 10)	*p* Value
Maternal age (year)	33.4 ± 5.1	32.8 ± 3.4	0.658
Gestational age at delivery (week)	39.1 ± 1.1	35.9 ± 2.2	0.583
Gravida	2.1 ± 1.4	1.8 ± 1.2	0.560
Blood pressure (mmHg)			
Systolic blood pressure	114.6 ± 10.5	151.4 ± 15.1	<0.001
Diastolic blood pressure	75.1 ± 7.5	94.6 ± 10.3	<0.001
Pre-pregnancy BMI (kg/m^2^)	23.6 ± 4.9	22.2 ± 3.7	0.375
Birthweight (g)	3435 ± 457.0	2199.8 ± 654.8	<0.001
Small gestational age newborn (*n*)	1	10	0.023

**Table 2:** Primers used for gene amplification

GREM1	Sense-gccagtgcaactctttctac	EPHB3	Sense-tctcccagattgtcaatacc
	Antisense-tcttggtaggtggctgtagt		Antisense-ctgtcgtgaaggttgtgtaa
VEGFA	Sense-actgaggagtccaacatcac	AMOT	Sense-ggaccacatcgtttgtctat
	Antisense-tctgcattcacatttgttgt		Antisense-aggatctgaatgggagtttt
THSD7A	Sense-cttgtaacccaccgtgtagt	ANGPTL4	Sense-gctggacagtaattcagagg
	Antisense-caagggtgctgttagaagac		Antisense-gtgatgctatgcaccttctc
SEMA5A	Sense-tcaccctgctcgtctatact	FGF2	Sense-ggcttctaaatgtgttacgg
	Antisense-gtggttggttatgctggtat		Antisense-ttatactgcccagttcgttt
GBX2	Sense-tagagaaggagttccactgc	GAPDH	Sense-atggggaaggtgaaggtcg
	Antisense-ttctggaaccagattttcac		Antisense-ggggtcattgatggcaacaata

## Data Availability

The data that support the finding of this study are available from the corresponding author upon reasonable request.
